# A Case of Infective Endocarditis and Spinal Epidural Abscess Caused by* Streptococcus mitis* Bacteremia

**DOI:** 10.1155/2017/7289032

**Published:** 2017-09-05

**Authors:** Victoria S. Byrd, Attila S. Nemeth

**Affiliations:** ^1^Case Western Reserve University School of Medicine, Cleveland, OH, USA; ^2^Department of Medicine, Louis Stokes Cleveland VA Medical Center, 10701 East Blvd. 111W, Cleveland, OH 44106, USA

## Abstract

A 57-year-old man presented with abdominal pain, hematemesis, and melena. He reported taking high-dose ibuprofen for back pain and drinking several 24-ounce beers daily. Examination was remarkable for icteric sclera, poor dentition, tachycardia, and crescendo-decrescendo murmur at right upper sternal border, radiating to the carotids. Labs revealed leukocytosis, anemia, thrombocytopenia, and elevated liver function tests and INR. Endoscopy demonstrated antral ulcers, duodenitis, and esophagitis. Blood cultures were obtained and broad-spectrum antibiotics started; cultures later grew* Streptococcus mitis*, and antibiotic coverage was narrowed. Transthoracic echocardiogram (TTE) demonstrated aortic stenosis and regurgitation, but no vegetation. Repeat blood cultures were negative; however, the patient developed neurological symptoms concerning for cauda equina syndrome, and MRI revealed epidural abscess. Emergent decompression could not be performed as the patient developed hematemesis and required intubation. Transesophageal echocardiogram (TEE), initially deferred due to friable esophageal mucosa, was performed and revealed small aortic valve vegetation. Poor oral hygiene was felt to be the probable source of the patient's* S. mitis* bacteremia, epidural abscess, and infective endocarditis. The patient's neurological symptoms resolved without intervention and remaining teeth were extracted. This case demonstrates that* Streptococcus mitis* can result in clinically significant bacteremia, particularly in immunocompromised patients, including chronic heavy alcohol users.

## 1. Introduction

*Streptococcus mitis*, while often characterized as a benign oropharyngeal colonizer, can result in clinically significant bacteremia. This is especially true in immunocompromised patients. Here, we present the case of a 57-year-old man whose only known risk factors for* S. mitis* bacteremia were very poor dentition and chronic alcohol use. Despite being rare,* S. mitis* bacteremia is associated with significant morbidity and mortality. In this case, the patient's bacteremia resulted in a classic presentation for infective endocarditis and a spinal epidural abscess.

## 2. Case Presentation

A 57-year-old man with past medical history significant for hypertension, coronary artery disease, hepatitis B and C, chronic low back pain (LBP), and polysubstance abuse (alcohol, cocaine, tobacco, and remote intravenous (IV) drug use) presented with diffuse abdominal pain, periodic hematemesis, and melena for two weeks. The patient described the abdominal pain as a fullness. The patient reported that his LBP had been worse over the past two weeks and he had been taking high-dose ibuprofen (800 mg) six times a day. He reported a history of drinking six 24-ounce beers a day but stated that he had cut back over the past several weeks; his alcohol consumption at the time of presentation was three 24-ounce beers a day.

The patient's vitals were temperature of 37.2 degrees centigrade, pulse 106 beats/min, blood pressure 107/68 mmHg, and respiration 20 breaths/min. On physical examination, he was disheveled and malodorous, with slightly icteric sclera. He had very poor dentition; most of his teeth were missing or broken or contained dental caries. Cardiac exam was significant for tachycardia, regular rhythm, and 2/6 crescendo-decrescendo murmur at the right upper sternal border with radiation to the carotids. His lungs were normal. His abdomen was slightly distended but otherwise normal. Rectal examination demonstrated melanotic stool. Neurologic examination was nonfocal. He had a leukocytosis of 19.98. Hemoglobin (HGB) was 9.8; baseline was 14. Platelets were 88. Basic metabolic panel was significant only for potassium of 5.6 (hemolyzed). Liver function test demonstrated AST of 75, ALT of 167, alkaline phosphatase of 142, and total bilirubin of 4.3. Lipase was 491. INR was 1.90. Chest X-ray demonstrated hyperinflated lungs. CT chest/abdomen/pelvis demonstrated esophagitis, portal hypertension, cirrhosis, and pyloric thickening concerning for peptic ulcer disease. Two blood cultures were obtained and the patient was empirically started on piperacillin/tazobactam and vancomycin for broad coverage. He was then admitted to the ward. His MELD score was 19 on admission.

A nasogastric tube was placed and 350 mL of coffee ground fluid was lavaged. Gastroenterology was consulted for an esophagogastroduodenoscopy, which demonstrated duodenitis in the duodenal bulb, a few ulcers and erosions in the antrum, and severe esophagitis in the middle third and distal third of the esophagus. Blood cultures from admission grew* Streptococcus mitis*, which was sensitive to vancomycin and cefepime but had intermediate susceptibility to penicillin. Infectious disease (ID) was consulted and recommended starting the patient on ceftriaxone for four weeks. A transthoracic echocardiogram demonstrated moderate aortic stenosis with new moderate aortic regurgitation, but vegetation was not observed. Transesophageal echocardiogram (TEE) was deferred due to friable esophageal mucosa. Repeat blood cultures were negative.

During this admission, the patient developed new right lower extremity numbness and an episode of bowel incontinence, and an MRI lumbosacral (LS) spine was obtained due to concern of acute cauda equina syndrome. It demonstrated a 5.1 cm × 1.8 cm × 1.1 cm epidural abscess. Neurosurgery was consulted for possible emergent decompression. However, before decompression could be performed, the patient again developed hematemesis and was transferred to the MICU. He was started on octreotide and a proton pump inhibitor. Repeat HGB was 7.3. He was subsequently intubated for airway protection. He finally underwent a TEE, which demonstrated small aortic valve vegetation and severe aortic insufficiency. After stabilization, neurosurgery felt the patient was too high-risk for decompression. Repeat MRI LS spine demonstrated stable disease and his neurologic symptoms resolved without intervention, so neurosurgery recommended observation. Cardiac surgery also felt the patient was too high-risk for aortic valve replacement. CT facial bones did not demonstrate any dental abscesses. Nonetheless, poor oral hygiene was felt to be the most likely source of infection. According to ID's updated recommendations, the patient completed a six-week course of IV ceftriaxone. The patient's remaining teeth were extracted, as his dentition was severely impaired and it was felt that more conservative approaches would not be sufficient. He recovered without event.

## 3. Discussion

*Streptococcus mitis* is a member of the viridans group streptococci (VGS) family, which colonizes all surfaces of the oropharynx [1]. While normally considered commensal organisms of the oropharynx, viridans streptococci can escape from this habitat, especially in immunocompromised patients such as those with febrile neutropenia (FN). When this occurs, mortality ranges from 6% to 30% [2]. This case demonstrates the severe infectious complications that can result when* S. mitis* breaks out of its role as a commensal organism and becomes pathogenic. Interestingly, the patient in this case was not neutropenic. However, his chronic alcohol use may have predisposed him to this bacterial infection [3].

Of the VGS organisms,* S. mitis* is particularly virulent [4]. In a cohort of 118 cancer patients with VGS bacteremia,* S. mitis* was the most commonly involved organism, accounting for 68 cases. It was significantly more likely to cause primary bacteremia than other VGS species and was associated with more severe bacteremia [4]. In recent studies conducted in the UK, the rate of bacteremia due to* S. mitis* exceeded that due to group A or B streptococci [5]. Importantly, the* S. mitis* strains isolated from bacteremic patients in these studies frequently had resistance to common antibiotics [5]. In addition to bloodstream infections,* S. mitis* has been implicated in myriad diseases including dental caries, eye infections, meningitis, and pneumonia [5]. While* S. mitis* is a leading cause of infective endocarditis (IE) among the oral streptococci, to our knowledge, this is only the second case in which spinal epidural abscess (SEA) has been reported in association with* S. mitis* IE [6]. SEA is a rare complication of VGS-related IE in general; only eleven cases of vertebral osteomyelitis complicating VGS endocarditis have been previously reported [7].

Patients who develop infectious complications of* S. mitis* are most often infected with their own commensal strains [2]. The same strategies that allow* S. mitis* to colonize the oropharynx in healthy individuals serve as virulence factors in immunocompromised patients, promoting the development of bacteremia, IE, and other complications [5, 8]. Platelet-binding proteins encoded by phages represent an important virulence factor contributing to the pathogenesis of* S. mitis* endocarditis; it is likely that these same proteins promote primary attachment to oral mucosal surfaces in healthy individuals colonized by* S. mitis* [5, 9]. In addition,* S. mitis* evades the host immune system via clonal and antigenic diversity leading to frequent turnover of different strains, and by producing an IgA protease, both factors that facilitate colonization but likely also contribute to the pathogenesis of* S. mitis* infection. The majority of identified virulence factors present in the* S. pneumoniae* genome are also present in that of* S. mitis*; this includes several putative adhesins, as well as a capsule locus that may enable evasion of the host immune system as it does in* S. pneumoniae*. It is not known why the presence of these genes does not allow* S. mitis* to overwhelm the healthy human immune system the same way* S. pneumoniae* does. Nevertheless,* S. mitis* represents a significant danger to immunocompromised patients [5].

The patient in this case was a chronic heavy alcohol user (defined by the National Institute of Alcohol Abuse as >14 drinks per week for men) with a past medical history of untreated hepatitis C, as well as alcoholic liver disease and other substance abuse [10]. According to Szabo and Saha [3], alcohol use impacts both the innate and the adaptive immune responses, resulting in subclinical immunosuppression. A secondary insult such as bacterial or viral infection, or other tissue damage, is typically required for the immunosuppression to become clinically relevant. The tissue damage associated with this patient's poor oral hygiene, in combination with* S. mitis* colonization of his oropharynx, could have provided the secondary insult that allowed* S. mitis* to become an infectious pathogen of multiple organs in this individual. Oral bacteria are shed into circulation each time a person chews or brushes their teeth; however, in patients with poor oral hygiene, inflammation and ulceration of gingival tissue surrounding teeth cause proliferation and dilation of the periodontal vasculature, providing an additional entry site for VGS and other organisms [11–13]. These risk factors can be ameliorated by improved oral hygiene and gingival health. Therefore, good oral hygiene habits are likely to reduce the risk of IE and other complications of VGS bacteremia and may have prevented the development of* S. mitis* endocarditis and epidural abscess in the patient described in this case.
